# Implementation of a Care Pathway for Primary Palliative Care in 5 research clusters in Belgium: quasi-experimental study protocol and innovations in data collection (pro-SPINOZA)

**DOI:** 10.1186/s12904-015-0043-x

**Published:** 2015-09-28

**Authors:** Bert Leysen, Bart Van den Eynden, Birgit Gielen, Hilde Bastiaens, Johan Wens

**Affiliations:** Department of Primary and Interdisciplinary Care, University of Antwerp, Universiteitsplein 1, Antwerp, Wilrijk 2610 Belgium; Centre for Palliative Care Sint-Camillus, Oosterveldlaan 24, Antwerp, Wilrijk 2610 Belgium; InterMutualistic Agency, Tervurenlaan, 188/A, Brussels, 1150 Belgium

**Keywords:** Data linkage, Health services research, Palliative care, Primary health care, Surveys

## Abstract

**Background:**

Starting with early identification of palliative care patients by general practitioners (GPs), the Care Pathway for Primary Palliative Care (CPPPC) is believed to help primary health care workers to deliver patient- and family-centered care in the last year of life. The care pathway has been pilot-tested, and will now be implemented in 5 Belgian regions: 2 Dutch-speaking regions, 2 French-speaking regions and the bilingual capital region of Brussels. The overall aim of the CPPPC is to provide better quality of primary palliative care, and in the end to reduce the hospital death rate.

The aim of this article is to describe the quantitative design and innovative data collection strategy used in the evaluation of this complex intervention.

**Methods/Design:**

A quasi-experimental stepped wedge cluster design is set up with the 5 regions being 5 non-randomized clusters. The primary outcome is reduced hospital death rate per GPs’ patient population. Secondary outcomes are increased death at home and health care consumption patterns suggesting high quality palliative care.

Per research cluster, GPs will be recruited via convenience sampling. These GPs -volunteering to be involved will recruit people with reduced life expectancy and their informal care givers. Health care consumption data in the last year of life, available for all deceased people having lived in the research clusters in the study period, will be used for comparison between patient populations of participating GPs and patient populations of non-participating GPs. Description of baseline characteristics of participating GPs and patients and monitoring of the level of involvement by GPs, patients and informal care givers will happen through regular, privacy-secured web-surveys.

Web-survey data and health consumption data are linked in a secure way, respecting Belgian privacy laws.

**Discussion:**

To evaluate this complex intervention, a quasi-experimental stepped wedge cluster design has been set up. Context characteristics and involvement level of participants are important parameters in evaluating complex interventions. It is possible to securely link survey data with health consumption data. By appealing to IT solutions we hope to be able to partly reduce respondent burden, a known problem in palliative care research.

**Trial registration:**

ClinicalTrials.gov Identifier: NCT02266069.

## Background

### Importance of primary palliative care

Although palliative care pioneers had the desire to give a dignified death to all people, their efforts focused mainly on symptom control in cancer patients. Nowadays, a more comprehensive, community-oriented, population-based and public-health approach is developed by the palliative care community [1] and supported by the World Health Organization [2].

This comprehensive approach cannot be realized by palliative care specialists alone. General practitioners (GPs) express their interests in being involved in high quality palliative care for all people aiming to stay at home in their last phase of life [3]. This requires training the GPs in (generalist) palliative care, and offering them advice from a palliative care specialist when needed [4, 5]. Quill & Abernethy describe which roles both parties should take in this collaboration [6]. Well-functioning second-line palliative care networks who are collaborating with GPs and go to a patient’s home only on occasion have proven to lead to desirable outcomes [7, 8].

Good inter-professional collaboration between specialized palliative care teams and primary care professionals often leads to workplace learning, in which both parties can absorb each other’s expertise and experience [9].

### Challenges and opportunities for palliative care research in primary care

Several challenges are known in both palliative care research and primary care research. The key challenges for palliative care research are mainly related to the functional decline and suffering experienced by palliative care patients, leaving little room for performing extra tasks like participation in research. There is also the already high care burden for family members and the gate keeping function of health care professionals willing to protect patients and their family from respondent burden [10].

The key challenges in primary care research are that primary care teams are geographically dispersed, with per practice a small amount of patients possibly suitable for any study. Furthermore, every primary care practice participating in research has to go through four stages, with their own challenges: from (1) the agreement of the professional to participate to (2) actual participation of professionals, (3) patient agreement and (4) finally patient data collection [11].

Palliative care research in the primary care setting is thus expected to face double challenges: the gate keeping role of practitioners is suspected to be even stronger here [12]. Advances in electronic management of routine clinical data could have important implications for recruitment if concerns about confidentiality can be satisfactorily addressed. Both for observational and cohort studies and for trials requiring the consent of individual participants, the electronic health record could enable researchers to follow participants more easily followed over time [11]. These ideas lead to the concept of the Learning Health Care System, in which routinely collected clinical data inform research and practice-based research informs on its turn clinical service [13, 14].

### Setting

In Belgium, community-based specialist palliative care teams support GPs and other primary care workers confronted with complex palliative care situations. Palliative care networks (PCNs) have the assignment to teach health care professionals and to inform lay-people about palliative care principles, to teach and coach palliative care volunteers and to collect analyzable data on palliative care in their territory. Related to these networks, palliative home care teams, including experienced physicians, nurses, psychologists and/or social workers, advise primary care professionals dealing with concrete palliative care patients at home or in residential care settings (e.g. nursing homes and institutions for people with disabilities). Palliative home care teams aim to complement the generalist palliative care approach of primary care workers by specialist palliative care activities [7, 8].

For the scope of this article, it is also relevant to know about some practicalities of the Belgian health care system. Every person with a legal stay in Belgium has a National Registry Number (NN). This NN can be used to uniquely identify all people entering the health care system. All data on reimbursed health care consumption are linked to this personal NN. Health insurance is organized by 7 different health insurance funds (HI funds). Every person with a legal stay in Belgium is obliged to choose membership of 1 of those HI funds. Primary healthcare is mainly paid fee for service of which 75 % (or more in specific cases) is reimbursed afterwards by the HI funds. These institutions are independent organizations receiving their main income from the National Institute for Health and Disability Insurance (NIHDI). The different HI funds constitute the InterMutualistic Agency (IMA) to bring together electronically all the health care consumption data from all Belgian inhabitants for research purposes.

All registered health care professionals have their own NIHDI-number, comparable to NN for citizens. Most Belgian GPs work in a mono-disciplinary GP practice and on a fee-for-service basis. Many GPs work in single-handed practices. GPs are increasingly organizing themselves in group practices, which are sometimes supported by paramedical personnel and are seldom organized in a forfeit payment system. In 2012, 2,7 % of all Belgian patients were served by practices in this forfeit payment system, mostly multi-disciplinary group practices [15]. All people are free to choose their GP and GPs are free to accept or refuse patients. Because of this freedom of choice, there are no inscription lists, complicating practice denominator calculations. A financial measure with small benefits for both GPs and patients is encouraging patients’ fidelity towards the GP chosen by the patient. Professional guidelines on different (primary care) topics were compiled, published and updated regularly but are not compulsory.

GP circles, GP associations at meso-level, are important partners for change in the Belgian general practice landscape. These professional associations unite the local GPs, mostly 70 to 100 GPs. The two main goals of a local GP-circle are to organize out-of-hours primary care services and continuing medical education.

### The Care Pathway for Primary Palliative Care (CPPPC)

The development of the CPPPC should be seen in the context of trying to fulfill the educational needs of primary health care professionals in ‘basic’ palliative care skills [6]. The palliative care community has bad experiences with another Care Pathway, the Liverpool Care Pathway [16]. Of the 44 recommendations of its independent evaluation report [17] the most relevant for this project is number three: *“The name ‘Liverpool Care Pathway’ should be abandoned, and within the area of end of life care, the term ‘pathway’ should be avoided. An ‘end of life care plan’ should be sufficient for both professionals and lay people.”*

Still, our research group decided to keep the name of the intervention as a Care Pathway. Currently, there is a movement to integrate patient-centeredness more explicitly into the development, implementation and evaluation of care pathways [18, 19] – this CPPPC could be an example of how to (try to) do that, because of the importance it gives to the preferences of individual patients included in the Care Pathway.

The Palliative Care Research Group of the University of Antwerp developed and pilot-tested this care pathway in collaboration with the Flemish Federation of Palliative Care and the Belgian Dutch Care Pathway Network, with funding of the Flemish Government [20, 21]. The CPPPC is inspired by the philosophy of the 7C’s of the Gold Standard Framework [22]. Its components are summarized in Table 1.

**Table 1:** Components of the Care Pathway for Primary Palliative Care

1. Early identification of patients eligible for palliative care, using the Surprise Question [21] and/or the Supportive and Palliative Care Indicator Tool (SPICT) [28]
2. Early assessment of patient’s needs and wishes
a. Assessment of performance status, using the Palliative Performance Scale (PPSv2) [22, 23]
b. Assessment of the patient’s needs in the biological, psychological, social and existential aspects of his or her life
c. Advance care planning
3. Interdisciplinary discussion
4. Action: delivering palliative care
5. Registration in a ‘palliative care pathway’ file, common for all team members
6. Follow-up by the team, recognizing the different stages in the palliative continuum: the ‘early palliative’ stage (the patient cannot be cured anymore), the ‘transitional’ stage (the last months) and at last the ‘dying’ stage (the last few days)

GPs interested in implementing the CPPPC are taught about the four pillars in the development of care pathways: (1) research evidence, (2) clinical expertise, (3) local organization of care and use of tools and resources and (4) patient preferences [23]. The CPPPC is presented to GPs as bringing the first two pillars. Each GP should attribute the two other pillars, i.e. how he or she organizes the delivery of palliative care and his or her perception of how to handle concrete patient and their relatives.

For the implementation study we will be performing, the CPPPC focuses on the following aims:

1. Early identification of palliative care patients: GPs are asked to answer the “surprise question” (“Would you be surprised if the patient would die within a year?”) aiming to comprise a list of people with reduced life expectancy [24]. It is expected that this step will lead to earlier identification of palliative care patients than happens in usual care [25].
2. GPs introducing Advance Care Planning to these early palliative care patients: it is only when GPs share a certain awareness with the patient about the need for discussion of end-of-life care issues that GPs are considered to be able to start discussing Advance Care Planning with patients. To facilitate this step, recommendations for breaking bad news are taught in the educational sessions and are available at www.pro-spinoza.be in Dutch and French. It is expected that this step will lead to more awareness of both professionals, patients and family members of end-of-life care goals and that these goals will be more often attained.
3. Delivery of high quality palliative care: GPs are expected to deliver optimal quality of palliative care which could be achieved by three instruments suggested by the CPPPC: (1) the ‘palliative care pathway’-file, (2) the Palliative Performance Scale (PPSv2) [26], and (3) a manual on primary palliative care in Belgium, all available at www.pro-spinoza.be in Dutch and French. This ‘palliative care pathway’ has been designed to facilitate communication within the team of health professionals around the patients and between the health care team, the patients and the informal care givers, including among others a page listing issues on physical, psychological, social and existential issues within palliative care. The PPSv2 can help to detect milestones in prognosis: for instance, in cancer patients, it is known that a PPSv2 score of 60 % is correlated with a mean survival time of 92 days [27]. It is expected that this step will lead to higher patient and informal caregiver satisfaction, reduced number of hospital admissions as well as length of hospital stay in the last year of life besides a reduced hospital death rate.
4. Ensuring high quality care for the dying and for the bereaved: GPs are taught about warning signs (PPSv2 score of 20 % or lower) for the dying phase of patients and about good clinical practice in palliation. It is expected that this step will lead to a higher level of satisfaction with care for the dying and the bereaved.

### The implementation of the CPPPC in five clusters in Belgium

After the development of the CPPPC, the next step is to establish and document the implementation of this care pathway. The implementation was to be rolled out in five clusters.

Using a stepped-wedge cluster design, the authors will be able to closely guide the starting-up of the implementation of the CPPPC in all five clusters. The implementation scenario per cluster is summarized by Table 2, but can be adapted to the specific needs of each cluster.

**Table 2:** Implementation scenario per region

1. Finding a palliative care network willing to participate
2. Asking the GP circles within the territory of the palliative care network to promote this project to their member GPs
3. Organizing a ‘kick-off’ workshop and other educational sessions to motivate individual GPs to participate in this project, explaining the CPPPC and training the GPs to obtain an informed consent of palliative care patients
4. Organizing an inter-professional platform where representatives of both the palliative care network and the GP-circles meet every six months to evaluate the regional implementation strategy
5. Inviting representatives of the inter-professional platform of the region to an interregional platform every year, to enable them to learn from the experiences of the other regions

### The evaluation of the implementation of the CPPPC

The overall aim of this study is to evaluate whether the regional implementation of the CPPPC leads to an improved quality of palliative care in a region, seen from different perspectives: (1) of the people with limited life expectancy and their informal caregivers, (2) of GPs and (3) of the NIHDI as the national health care financing organization.

The specific objectives of this study are (1) a reduction of hospital death rate (primary outcome), (2) directing use of services during the last year of life towards quality-of-life (secondary outcome), (3) monitoring quality of life besides quality of care as perceived by patients, informal care givers and GPs (descriptive), (4) monitoring the level of implementation of the CPPPC by the GPs (descriptive) and (5) understanding in which circumstances (how and why) the implementation of the CPPPC works or does not work.

The theoretical framework guiding this evaluation study, is the framework of Grol and Wensing, describing five areas with possible barriers and facilitators for implementation of innovations in the health care system: the innovation itself, the targeted patients, the individual professionals, the social context, the organizational context, and the economic and political context [28].

Farquhar et al. state that a mixed methods study design is appropriate to develop and evaluate the implementation of complex interventions in palliative care [9]. The CPPPC being such a complex intervention, it has been decided to triangulate quantitative and qualitative research methods to evaluate this Belgian quality improvement project in primary palliative care.

### Aims of this article

For this methodological article, the focus is on the first four specific objectives of the study. In order to be clear on the quantitative methods used in this study, measurements of process and outcome indicators for the implementation of the CPPPC on GP, patient and informal care giver level are explained. In order to encourage other palliative care researchers to use large data sets linked with survey data, the privacy-respecting data handling procedures are explained.

The qualitative methods (focus groups, interviews, document analyses) used to obtain the fifth specific objective of the study will be explained in a future article.

## Methods/Design

### The stepped wedge cluster design (quasi-experimental)

For this study, a cluster design with a “stepped wedge” approach was chosen. This type of trial design involves sequential roll-out of an intervention to clusters over a number of time periods. The order in which clusters start the intervention is determined at random. In the end, all clusters will have started the intervention. This trial design is particularly interesting 1) when a certain intervention cannot be started simultaneously in all clusters, for instance because of logistical or financial reasons and 2) when researchers want to prevent ethical objections arising from withholding an intervention anticipated to be beneficial [29].

Five clusters will be involved (Table 3). The implementation of the CPPPC will be initiated in all clusters, in a stepped way. Ideally, to reduce bias, the different clusters need to be randomized from the start of the project. However, in this particular study, it was impossible to randomize the order of the regions from the beginning. Not all 5 clusters were well defined at the start of the project. Whenever we found a region’s palliative care network ready to participate, we offered that region the choice to start in the next phase, regardless of any preferences as a research team. That is why this study should be called a quasi-experimental study based on a stepped wedge cluster design.

**Table 3:** Characteristics of the five participating clusters, in chronological order of planned start [.]

Cluster	Official language	Nr of inhabitants [46]	PCN/Palliative home care teams situation	GP circles
1: Zone of Antwerp	Dutch	765.470	One legal entity	12, of which 3 not completely in the territory
2: Zone of Mons	French	572.979	One legal entity	8, of 3 which not completely in the territory
3: Brussels Capital Region	Dutch/French	1.163.486	1 PCN, 1 Dutch-speaking and 3 French-speaking palliative home care teams	1 Dutch-speaking + union of 19 French-speaking
4: Province of Limburg	Dutch	856.280	PCN and palliative home care teams separate, residing in the same building	17, of which 1 not completely in the territory
5: Province of Namur	French	484.737	One legal entity	8, of which 2 not completely in the territory

### Stepped wedge cluster procedure

It was decided to have phase intervals of six months, starting from the 1st of April 2013. The first phase is a ‘usual care’ phase for all the clusters. Every next phase, the intervention will be started in one cluster. The last intervention phase (phase 6) lasts until the 31st of March 2016. After this last date, no more ‘interventions’ on behalf of the study will be undertaken: neither meetings or educational sessions or recruitment of GPs and patients for the study.

A post-intervention data collection period for included patients will follow. This period (phase 7) lasts for 33 months, enabling the authors to cope with the effect delay of the intervention: patients will probably not all die in the same phase they were included in the study. Data collection will stop the 31st of December 2018.

**Fig. 1:**
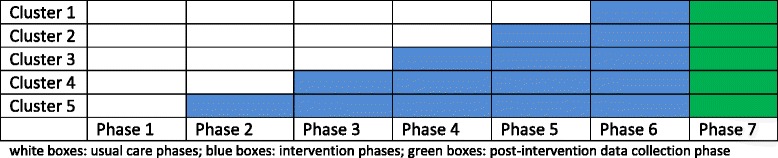
Stepped wedge cluster design applied in this study

A time schedule of the ‘stepped wedge cluster design’ is shown below (Fig. 1).

### Study population

A comparison will be made between the patient populations of participating and non-participating individual GPs, located within the territories of the participating PCNs(see 2.5). Data of participating patients will be used to monitor level of implementation and quality of care (see 2.6).

Patients of which data will be available correspond to these criteria:

1. To have a GP residing within the territory of a participating PCN, or to be domiciled there if the patient does not have a GP;
2. To have died a ‘non-sudden death’ in the study period (data of patients for comparison of GPs’ patient populations) and/or have a ‘positive surprise question’ according to their GP [20] (data of participating patients for the monitoring of quality of care);
3. To be at least 45 years old at the moment of inclusion.

### Intervention and research procedures

GPs interested in implementing and evaluating the CPPPC, are trained to implement the CPPPC (see The Care Pathway for Primary Palliative Care (CPPPC)), to fill web-based questionnaires reflecting the components of the CPPPC and to inform and motivate patients and their relatives to take part in the study.

For this evaluation study, the CPPPC was operationalized as follows:

1. Determination of a practice denominator: participating GPs will note how many patients are consulting them in a defined time period of 10 consecutive weekdays. For every person on this ten days practice denominator list, GPs are asked to answer the “surprise question” aiming to comprise a list of people with reduced life expectancy [24].
2. Identification of patients eligible for the study: during, but also after the determination of the practice dominator, GPs are expected to identify palliative care patients in an early way. For this study, only patients of 45 years or older are eligible.
3. Introduction of Advance Care Planning: participating GPs are asked to introduce Advance Care Planning to eligible patients. This will happen with or without an explicit understanding of the palliative care situation by the palliative care patient, depending of the GPs understanding of the information needs of the patient. If deemed appropriate by the GP, the GP will ask the patient to sign the informed consent to take part in this study. In the CPPPC training, it is stressed that (1) patients found eligible can, but are not obliged to be included and (2) that GPs should use their common sense, to make sure that both Advance Care Planning and the study are introduced in a sensitive way.
4. Delivery of high quality palliative care: GPs are expected to deliver optimal quality of palliative care. GPs are taught about the three instruments suggested by the CPPPC as mentioned in 1.4, but in a Belgian healthcare tradition use of these instruments is not compulsory. GPs are asked to fill monthly questionnaires on the PPSv2 of included ‘focus’-patients. Patients and informal care givers are asked to fill monthly or sometimes weekly questionnaires on the holistic aspects of the illness trajectory and patient experience of health care. More details are described in 2.6.
5. Ensuring high quality care for the dying and for the bereaved: GPs are taught about warning signs (PPSv2 score of 20 % or lower) to detect their patients’ dying phase and about good clinical practice in care for the dying. GPs are asked to be creative in providing continuing (bereavement) care for the family members when the patient has died. One month after the death of an included patient, both the GP and the informal care giver will be asked to fill a questionnaire on the quality of dying and on bereavement care.

### Outcome measures (health care consumption data)

The IMA uses algorithms to identify patients for the study population and to deliver data relevant to the research questions. These data identified by the NN of all patients in the study population (see 2.3) and NIHDI-numbers of their GPs will be coded by an innovative coding strategy, as explained more in detail in 2.9. This data collection will end the 31st of December 2018, two years and nine months after the end of Phase 6 (see Fig. 1).

#### Primary outcome: reduction of hospital death rate

The primary outcome of this study is a reduction of all hospital deaths from 50 % [30, 31] to 35 %. In Belgium, about half of people die in the hospital, including about 5 to 10 % of deaths in a palliative care unit [30, 31]. Through health care consumption data, it can be known which percentage of the deceased of a GP practice, in a certain period died at home, in a nursing home, in a hospital or elsewhere.

#### Secondary outcome: use of services

The secondary outcome is directing use of services towards quality of life in the last year of life. The ideal health care spending pattern is considered to be palliative home care (more contacts with GPs than with hospital doctors, …), with more use of symptomatic medication than of medication with a clear life prolonging intention and reduction of stressful diagnostic and therapeutic procedures. Indicators here, available by the IMA, are:

- Contacts of patients with primary care: GPs, home care nurses, physiotherapists
- Support of patients by palliative home care: palliative forfeit, palliative home care teams
- Emergency department visits with or without GP referral
- Length of stay in hospital, nursing home, or palliative care unit
- Use of medication with clear curative or life prolonging intent (e.g. statins)
- Use of symptom medication (e.g. morphine, antidepressants)
- Selection of diagnostic procedures: common lab tests, imaging procedures
- Selection of therapeutic procedures: blood transfusion, ascites paracentesis, etc.
- Health care consumption costs.

### Monitoring of implementation, quality of care and quality of life (GP and patient surveys)

Participating GPs, patients and relatives will fill periodical web-based surveys on illness trajectory and different aspects of care quality. These surveys will be collected via a secured electronic data collection system. All surveys have been drafted and piloted in Dutch, and have been back-and-forth translated to French; they are available on request.

To identify uniquely all patients involved, their NN numbers are used. To guarantee confidentiality of these personalized research data, the unique NN numbers will be coded electronically by eHealth, the health care data coding agency of the Belgian federal government (see 2.9 for more details). Doing so, re-identification of patients by the analysts is impossible.

The professional NIHDI number of participating GPs number is used for the data collection and coded by similar procedures during the data handling process, making re-identification of the health care professional impossible.

This data collection strategy will end the 31st of December 2016, nine months after Phase 6 (see Fig. 1).

#### Monitoring level of CPPPC implementation

On the regional level, the number of GPs reached by educational sessions and regional platform meetings will be measured, besides the quality of these educational sessions as perceived by attending GPs and the number of GPs interested to participate.

Participating GPs will be asked (1) to fill an 11-item baseline survey including personal characteristics and the 10 days practice denominator and (2) to include eligible patients for the study, i.e. being older than 45 years and having a ‘positive surprise question’ according to the GP [24]. For all included patients, the GP will fill a baseline survey on the patient’s medical condition based on the validated Supportive and Palliative Care Indicator Tool (SPICT)(version June 2013) [32]. To reduce the barrier of administrative overload for GPs, in this baseline survey the GP can choose whether a single-item survey (PPSv2) will be filled for this patient: if yes, this patient is called a ‘focus’-patient.

Per ‘focus’-patient, the GP fills a single-item survey (PPSv2) monthly, or weekly if the PPSv2 score is becoming 30 %, or less (when the patient gets weak and life expectancy decreases more) [33]. When a participating patient dies (‘focus’-patient or not), the GP will fill a 19-item survey on self-perceived quality of care delivered for this patient with a self-assessment for performance and two open questions. This survey was constructed by the research team to reflect the components of the CPPPC and has been minimally validated by informal peer review. The more steps are completed by a GP, the higher the level of involvement in the CPPPC is considered to be.

#### Monitoring quality of care and quality of life

Participating patients will receive a 14-item base-line survey to answer background questions on different psychosocial, cultural and spiritual issues.

Afterwards, every person involved in the study or an appointed relative will receive surveys about quality of life and quality of care periodically. This 26-item survey is an amalgam of adapted versions of the Palliative Outcome Scale (POS) [34] and (POS-S) [35], the NIVEL quality indicators for palliative care [36, 37] and a limited number of questions related to the 7 C’s of the Gold Standard Framework [22]. Several parts of this survey have been validated separately, but not the sum of it. The frequency for this questionnaire is monthly if the PPSv2 score is unknown or more than 30 %, and weekly if the PPSv2 is 30 % or less.

After the death of a participating patient, the appointed relative will fill a 5-item survey on perceived quality of care during and after the dying process.

### Sample size calculation

The average size of the patient population per active GP is estimated to be approximately 1000 [15]. With a mortality of 1 % per year [38], 10 patients per GP per year are expected to die, of which 9 are expected to die non-suddenly [39]. Pre-existing differences are expected to exist between the patient populations of GPs. For instance (although not found in literature), the age of GPs is expected to be positively correlated with the age of his patients, which is relevant for palliative care delivery. On a district level, the availability of health care services [40] and on a socio-cultural level the preferred use of health care services also influence patient outcomes [41]. Therefore, we assume in the following power calculations a relatedness between eligible patients within a GP patient population expressed by an intra-class correlation coefficient of 0.2 and an average of 9 eligible patients per GP.

To detect a difference of 15 % hospital death rate between the trained and the untrained GPs, a classic randomized control trial with two groups, taking into account an intra-class correlation coefficient of 0.2, would require a sample size about 440 GPs to reach 80 % statistical power. Thanks to the stepped wedge design implemented in this study, with 5 clusters, 5 steps and 1 baseline measurement, the required sample size of GPs is reduced to approximately 180, as calculated in a recently published formula [42]. The total number of GPs amenable in this study is approximately 3500. Hence, a participation rate of 5 % is required. This participation rate of 5 % is considered realistically achievable, according to Rogers’ Innovation/Adoption curve (15 % of all people are considered ‘innovators’ and ‘early adaptors’, adapting innovations fast) [43].

### Recruitment strategies

#### Recruitment of PCNs

Participating PCNs will have signed a collaboration agreement (convenience sampling). The role of PCNs in the study, as explained in Table 2, is to facilitate the local coordination of the project, (1) by bringing the research team in contact with local GP circles and local GPs interested in palliative care (research), (2) by organizing educational sessions for GPs and paramedics and (3) by supporting the logistics of both the quantitative and qualitative data collection.

Participating PCN are paid a small financial compensation for completion of these tasks.

#### Recruitment of GPs through educational sessions

A diversity of educational sessions is organized to inform local GPs (and local paramedics) within the territories of participating PCNs. A kick-off workshop is organized at the start of the intervention in every region (see Table 2). In this kick-off workshop, GPs and paramedics are invited to attend a 3-hours program discussing the highlights of the CPPPC (see 1.4). This kick-off workshop aiming for an audience of 50 to 100 participants is designed to help ‘spreading the news’ to all local primary health care professionals. This rather large-scale recruitment intervention is followed by many interventions of a smaller scale: Continued Medical Education sessions for GPs for 10 to 50 participants during 1 to 1,5 hours and GP office visits for mostly 1 to 10 GPs during 30 to 60 minutes. These small-scale educational sessions are ideal to have (group) discussions on the barriers and facilitators to deliver palliative care in general and to promote the CPPPC as a tool to improve palliative care practices.

GPs showing interest in the project, by attending these educational sessions about the CPPPC, are asked to participate in the study (convenience sampling). Participating GPs will have signed a collaboration agreement and will know that for the first questionnaire filled and for patients included for the project (see 2.6.1) they will be paid a small compensation fee.

#### Recruitment of patients and informal care givers

Participating GPs have received minimal training on informed consent taking for research purposes by the research team and are asked to include eligible patients (see 2.3) for follow-up of the intervention. GPs are taught about the importance of having the support of the patients’ informal care givers for the completion of the surveys. Therefore every patient is asked to appoint a relative for assistance. Patients are asked by their own GPs to sign the informed consent form. Patients and their informal care givers will not be rewarded financially for their participation.

### Linkage of health care consumption data and questionnaire data

Both data collection systems (questionnaires from GPs and people in the palliative care setting besides health care consumption data) will be linked securely allowing more in-depth analysis, taking into consideration all confidentiality regulations, as promulgated by the National Commission for the Protection of Privacy and its Sectorial Committee of Social Security and Health Care. Therefore, the Sectorial Committee had to give written approval in advance with all data handling procedures as used in this research project. (deliberation number SCSZG/13/251 dd. 19 November 2013) The acronyms used in this chapter refer to the figures showing the data linkage process.

#### Coding procedure for the health care consumption data: standard data trajectory of the IMA (see Fig. 2)

**Fig. 2:**
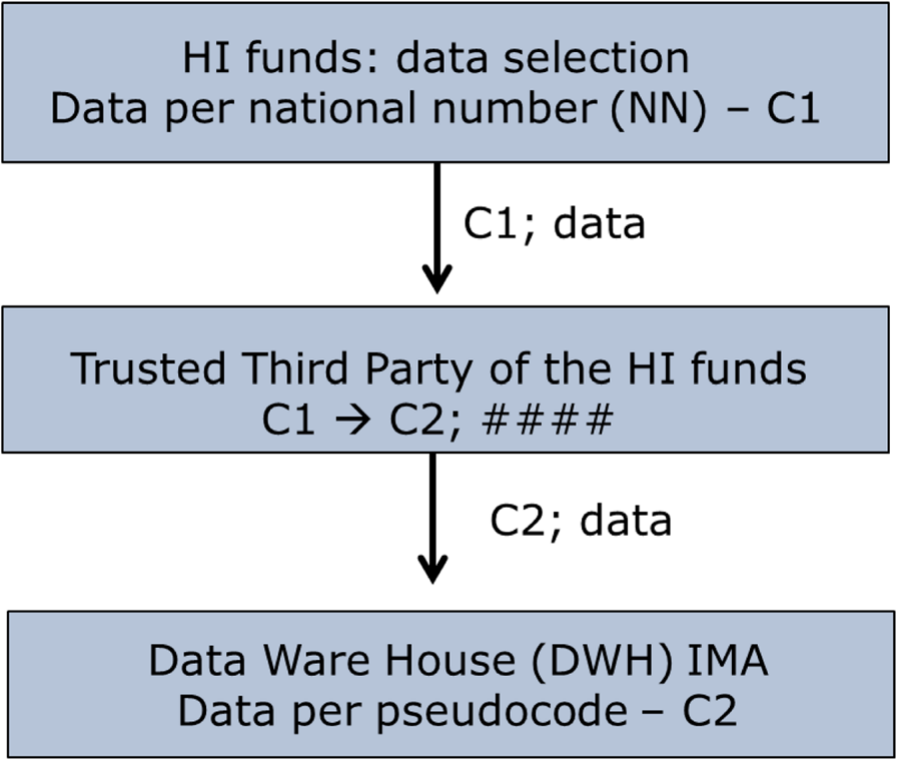
Coding and encryption: standard data trajectory of the IMA

First, at the level of the HI funds, the unique NN of all patients in the study population (see 2.3) will be transformed by the security officer to the patient-pseudo code C1. This C1 still is linked to the original health consumption data and will be sent to the Trusted Third Party (TTP) of the respective HI funds, which encrypts the data (####) while transforming C1 to another pseudo code C2. In the end, these encrypted data (###) paired with the C2 pseudo code are sent to the Data Ware House of the IMA. Here, all encrypted health care consumption data are stored in a secured way. This is the standard data trajectory of the IMA.

#### Selection of relevant health care consumption data for the prospective study

The TTP of the HI funds knows which C2 of the IMA-Data Ware House is linked to which C1. This is how the relevant IMA data (see 2.5 for more details) can be extracted by the TTP of the HI funds and stored in a separate sector of the IMA Data Ware House: the Project Data Ware House.

The IMA analyst will now categorize IMA-data and label the desired groups of GPs and patients(see 2.10 for more details). For this procedure, the IMA analyst works with research data linked to the unique project code and with the original NIHDI-number of the GPs of the research clusters. The original NIHDI-numbers are only coded in a later stage of the coding and linkage procedure because the original NIHDI-numbers are needed by the IMA-analyst to perform the pre-analysis.

#### Coding procedure for the questionnaire data (see Fig. 3)

**Fig. 3:**
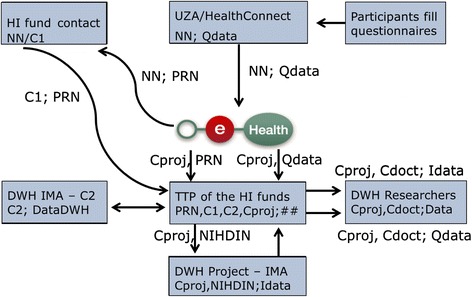
Data flow of patients having signed an informed consent (questionnaires available)

All data from the questionnaires (Qdata) initially are linked with the personal NN of patients. For GPs, the data are linked with their NIHDI number. Immediately after answering, this privacy-sensitive information is sent directly to eHealth, which will perform three actions.

First, a unique Project Random Number (PRN) to be used only in this specific data handling procedure is linked with the NN. The link between these two numbers will be sent to the security officers of the different HI funds. Here again, the unique NN will be transformed towards a patient pseudo code C1 and then sent to the Trusted Third party (TTP) of the HI funds.

Secondly, eHealth sends the link between the PRN and the project code, i.e. the patient-pseudo code which will ultimately be used for analysis (Cproj), to the TTP of the HI funds.

Finally, a third link between the project code (Cproj) and the questionnaire data (Qdata) is sent from eHealth to the TTP of the HI funds.

#### Linkage of questionnaire data and health care consumption data (see Fig. 3)

After pre-analysis, the IMA project database (Idata) is sent through the TTP of the HI funds to the authors’ Data Ware House which will be used to analyze all completely coded data, whether they come from the questionnaires (Qdata) or from the IMA (Idata). At this last stage, the original patient’s NNs are replaced by project codes (Cproj) and the doctor’s original NIHDI-numbers are replaced by doctor pseudo codes (Cdoct). Survey and IMA data will be linked with an estimated failure risk of max. 2-3 %.

#### Selection of relevant health care consumption data for the retrospective study (see Fig. 4)

**Fig. 4:**
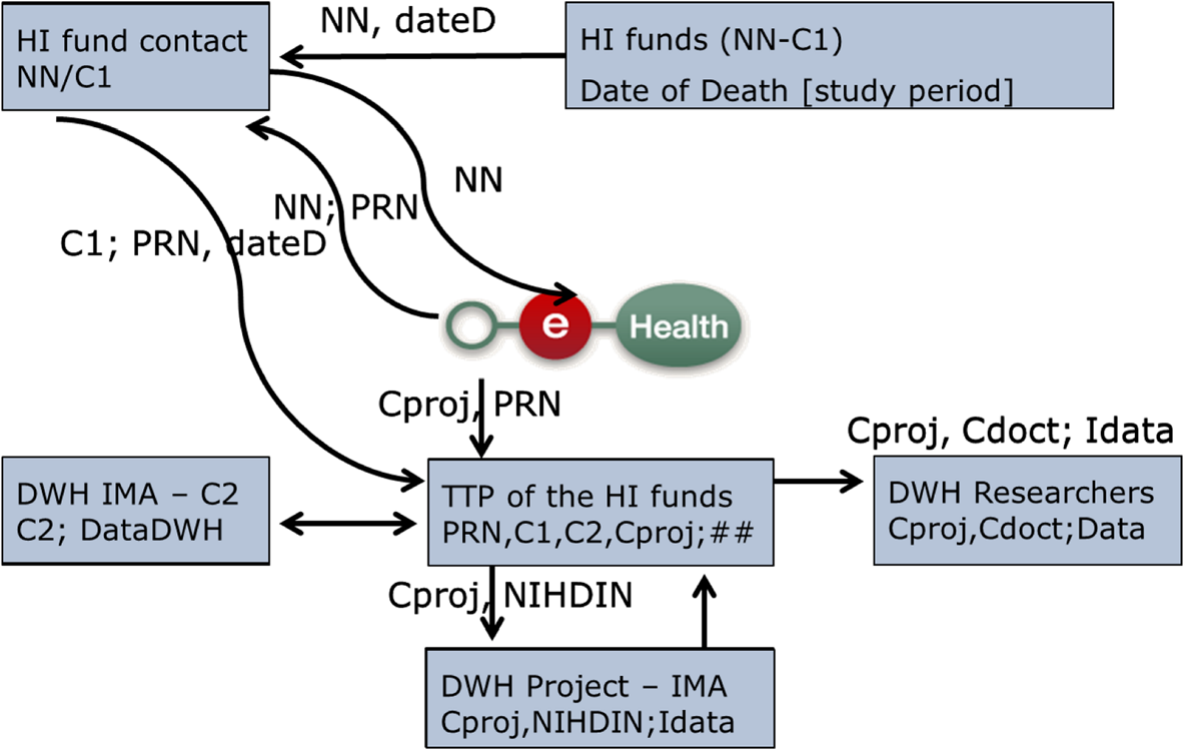
Data flow of patients not having signed an informed consent (surveys not available)

For patients not having signed an informed consent, the inclusion and first data flows are different and questionnaire data are off course not available. The inclusion will be done in the database of the HI funds as explained in “2.3 Study population”.

Of these people, the NN will be sent to the security officers of the HI funds. These officers will send the NN to eHealth. From then on, the data flow is very similar to the one of patients with an informed consent.

### Analysis

For the analysis of primary and secondary outcome measures, longitudinal and cluster analysis are to be used to compare data of patient populations of participating GPs vs. patient populations of non-participating GPs. If possible, the IMA will match included GPs with control GPs based on age category (per 5 years) and GP circle. If matching proves not to be realistically achievable, these characteristics of the GPs will be considered independent variables.

The data related to monitoring the implementation of the CPPPC and the quality of care and quality of life, are to be used descriptively to contextualize the primary and secondary outcomes related to participating GPs and patients. To be able to describe the GPs’ patient populations, data will also be available through the IMA on the patients’ age category, gender, month of death, disease category (cancer vs. non-cancer; presence of dementia) and socio-economic status.

### Ethical considerations

The study design has been approved by the Ethical Commission of the University of Antwerp (number 13/35/333, date: 7/10/2013) and by the Belgian Commission for the Protection of Privacy (statement SCSZG/13/251, date: 19/11/2013).

#### Stepped wedge cluster design

A thorough consideration of the ethical aspects of the stepped-wedge design is important, especially for community based research when ‘informed consent’ cannot be obtained on an individual level [44]. When it is assumed that the intervention is more likely to benefit the patient than to harm the patient, as is assumed for high quality palliative care, the advantage of the stepped wedge cluster design is that all clusters will have received the intervention in the end, while still comparing data between intervention and control groups.

#### Data retrieval after death

The health care consumption data are obtained with a ‘waiver of consent’, commonly used in cluster designs analyzing coded data on community-basis [45]. A waiver of consent is granted by an ethical committee if it is deemed ethically reasonable, that data of persons can be analyzed even without their explicit informed consent. This procedure is necessary here, because of two reasons: 1) these health care consumption data of deceased persons are very relevant for the evaluation of the CPPPC and 2) it is impossible to have an informed consent of all members of the target population. In this situation it is of utmost importance to preserve confidentiality of the data.

#### Informed consent procedures

Both GPs and patients have to sign that they agree with the research procedures, as explained above. GPs are supported by the authors to collect data and to make it to the next step in the procedures in a correct way, for both academic and humane aspects of recruiting patients and collecting data. If a GP or a patient wants to stop delivering data, one phone call to the PCN will be enough to do so.

#### Data handling procedures

In Belgium, every new data handling procedure involving privacy-sensitive data, for instance data linked with NN or NIHDI numbers for research, as is the case in this evaluation study, must be approved by the National Commission for the Protection of Privacy before use. This approval has been granted because the procedures rely on the safest data handling procedures available, as explained above.

This Commission for the Protection of Privacy does not only give advices on technical procedures, but also on the content of information to be transferred through the procedures.

## Discussion

A general discussion of the research protocol and its rationale is followed by a further discussion of the major advantages and disadvantages of collecting data by means of an electronic platform, how this study design tries to tackle the six key challenges in palliative care research and how this study design fits into the Learning Health Care System’s paradigm.

### General discussion

The CPPPC is a tool to improve palliative care practices, by starting palliative care earlier with the introduction of anticipatory care planning, and by providing comprehensive and interdisciplinary care for patients included in the Care Pathway. Its strength seems to be its combination of systematical assessments and patient-centeredness. The primary outcome for this study is a reduction of hospital-based deaths from 50 % to 35 % and the secondary outcomes are an increase of deaths at home and an increase of health care consumptions patterns suggesting high quality palliative care. To achieve these objectives, the challenge to be overcome seems to be the complexity of the CPPPC. That is why it is important to monitor GPs’ level of involvement in the study and the patients’ appreciation of the CPPPC .

To be able to evaluate the implementation of the CPPPC, a quasi-experimental stepped wedge cluster design has been set up. The main advantage of this trial design is the roll-out per time period in the different clusters, allowing the researchers more dedicated time per cluster for the start-up of the intervention. A limitation here is the fact that randomization of research clusters was impossible, because not all 5 clusters were known at the start of the project.

The recruitment strategies involving different hierarchical levels (PCNs and GP circles on the meso-level and GPs and patients and their informal cares on the micro-level) provide opportunities for the establishment of a research network, but are expected to be time-consuming.

Qualitative methods complementing the quantitative methods explained in this research protocol will be described in a future article.

### Major advantages and disadvantages of an electronic data collection system

The authors want to stress the innovative nature of the electronic data collection system. There are many advantages here. Data collection occurs securely and coded from the start onwards. The financial investment will probably be worth it, considering all costs and practical difficulties related to research performed in an analogue way: printing and sending questionnaires on paper by regular post, hoping that the questionnaires return to the research team in a well filled and readable way, using unique codes for linkage all the data arriving from different sources, and at last the manual input of data. All these aspects of research are bypassed by the electronic data collection system.

Off course, to find palliative care patients willing to fill web-based questionnaires is not an easy task, and to stay motivating them to continue is expected to even be harder. That is why the authors ask the GPs to motivate informal care givers of these palliative care patients to (support them to) fill the questionnaires. Still, data collection bias can be expected for ill patients and overburdened informal care givers are expected to be less likely to fill all surveys. Furthermore, older people (our target group) are less likely to have access to IT-facilities.

### Coping with challenges in palliative care research

According to Farquhar et al. [9], there are six key challenges in palliative care research which can be answered by mixed methods research. Table 4 describes how this study tries to tackle all six key challenges. Although there was attention for these key challenges while designing this study, recruitment of GPs and patients is still expected to be time-consuming.

**Table 4:** Challenges for palliative care research and solutions given here

The six key challenges	How this study tries to solve them
Recruitment	
- Varying definitions of palliative care among clinicians and so-called ‘gate-keeping’: clinicians and family members keeping patients from participation	- Suggesting a ‘clear’ starting point for palliative care, i.e. life-expectancy of one year; training the GPs in communication skills specifically to obtain an informed consent.
- Inability of patients to give informed consent	- By trying to recruit patient participants in the early palliative stage.
Attrition: missing data and drop-out	- By having complementary datasets. For instance, if a participating patient stops delivering data, the authors still have access to the health consumption data.
Differing disease categories	- Distinctions will be made related to disease category in the health consumption data and the baseline questionnaires.
Respondent burden	- The researched unit is not only the patient, but also the informal caregivers and the GP – the research team hopes that all three components of this triangle can encourage each other in the data collection process.
	- Only to start there will be little paperwork, afterwards, an email will have to be answered once a month.
	- If a patient, the informal care giver or the GP are tired of delivering data, they must contact the PCN to stop participating in the study; this allows the PCN or its related palliative home care team to help participants in clinical aspects, if appropriate and necessary.
Randomization: sometimes, randomization means denying an intervention to patients	- The stepped wedge cluster design allows the authors to implement the intervention in all clusters.
Outcomes	- A multitude of research methods lead to a prism of outcomes pointing to quality of palliative care. Validated questionnaires like POS and POS-S were combined and reduced to balance importance of outcome measuring with avoidance of respondent burden.

### This approach fits in the Learning Health Care System’s paradigm

This study uses routine health care consumption data to describe the possible effect of this intervention aiming to improve palliative care delivery in the community. This approach being similar to the concepts of the Transform Project [13], in which routine data in the primary care Electronic Health Records (EHR) are used to find patient EHR data for research purposes [12], fits into the ideal of the Learning Health Care System. Doctors and patients don’t have to do extra efforts to deliver useful data, while the researchers and the regulating bodies guarantee the privacy of these data. In the end, large-scale epidemiological and health-economic studies will be made possible in a feasible way [12].

## Discussion and conclusion

This article explains the quantitative methods used in the evaluation study of the CPPPC (the first evaluation study of a primary palliative care intervention in Belgium), and shows an innovative way of data collection and linkage in a secure way, making it more feasible for researchers to analyze large data sets from different sources. A summary of this article can be read in Table 5. It is hoped for, though not yet proven that the IT solutions used will partly reduce respondent burden, a known problem in palliative care research.

**Table 5:** Summary

What was already known on the topic:
1. Primary palliative care is an emerging field.
2. Recruitment for palliative care research and for primary care research is a difficult process.
3. Web-based questionnaires can be used for research purposes.
4. In Belgium, health consumption data are available for research through the InterMutualistic Agency.
What this article added to our knowledge:
1. Data linkage of personalized web-based questionnaires data and individual health consumption data is possible in a secure way with respect for confidentiality issues.
2. Electronic data coding algorithms allow researchers to use individualized but coded health care data for assessment of complex interventions, while respecting the privacy of included patients.
3. IT solutions could partly reduce respondent burden in health care research.

## Abbreviations

CPPPC: Care Pathway for Primary Palliative Care
IMA: Intermutualistic Agency
HI fund(s): Health insurance fund(s)
NIHDI: National Institute for Health and Disability Insurance
NN: National Registry Number
PCN: Palliative care network
POS: Palliative outcome scale
PPSv2: Palliative performance scale (version 2)
SPICT: Supportive and palliative care indicator tool
TTP: Trusted third party
